# Maternal and perinatal guideline development in hospitals in South East Asia: results from the SEA-ORCHID project

**DOI:** 10.1186/1478-4505-7-9

**Published:** 2009-05-08

**Authors:** Jadsada Thinkhamrop, Tari Turner, Sivasangari Subramaniam, The SEA-ORCHID Study Group

**Affiliations:** 1 Department of Obstetrics and Gynecology, Faculty of Medicine, Khon Kaen University, Khon Kaen 40002, Thailand; 2 Monash Institute of Health Services Research, Monash University, Monash Medical Centre, Clayton, Victoria, Australia; 3 Department of Obstetrics and Gynecology, Hospital Ipoh, 30990 Ipoh, Perak, Malaysia

## Abstract

**Background:**

Recognising the potential of clinical practice guidelines (CPGs) to improve practice, one of the strategies of the SEA-ORCHID project was to facilitate the development of evidence-based CPGs, and to support clinical staff in each of the four countries to build their skills in development of CPGs in the nine participating hospitals in Thailand, Malaysia, Philippines and Indonesia. This study was undertaken to investigate the impact of the SEA-ORCHID project on development of evidence-based CPGs.

**Methods:**

Data on the CPGs available to support maternal and perinatal healthcare were collected by SEA-ORCHID team members at each hospital before and after the intervention period of the project.

**Results:**

There were only a few evidence-based CPGs available in the SEA-ORCHID hospitals before the intervention period. After the intervention period, in the SEA-ORCHID hospitals in Malaysia and Indonesia there was no change in evidence-based CPG development activity in maternal and perinatal care. In Thailand and The Philippines there was a small increase in evidence-based CPG development activity in maternal and perinatal care.

**Conclusion:**

Despite the wide range of interventions to support evidence-based CPG development implemented in the hospitals participating in the SEA-ORCHID, very little change was seen in the development of evidence-based CPGs.

## Background

Each year more than half a million women die in and as a result of childbirth worldwide. Ninety-eight percent of these deaths occur in the developing world. For women in Asia the lifetime risk of maternal death is one in 65, a rate that is more than 25 times higher than it is for women in developed countries. [1] Rates of perinatal and infant death are also high in developing countries. Each year there are almost eight million stillbirths and early neonatal deaths, along with increased rates of morbidity such as low birth weight, birth asphyxia and infection. [2] These striking figures are a clear indication that work is needed to improve health care for mothers and infants in developing countries

The South East Asia Optimising Reproductive and Child Health in Developing Countries (SEA-ORCHID, ) project is a five-year collaborative project (2004–08) between four countries in South East Asia (Thailand, Malaysia, Philippines and Indonesia) and Australia funded by the Wellcome Trust and the Australian National Health and Medical Research Council.

By establishing a network of researchers and teachers of evidence-based health care across Thailand, Malaysia, Philippines and Indonesia, supported from Australia, SEA ORCHID aims to examine whether the health of mothers and babies in the four South East Asian countries can be improved by increasing the capacity for research synthesis and improving the implementation of effective clinical practices. [3]

The evaluation of the SEA-ORCHID project includes quantitative evaluation of change in clinical practice, health outcomes, research activity and guideline development or adaptation; combined with qualitative investigation of the barriers to and enablers of these changes.

One of the key ways in which effective practice can be implemented is through the use of evidence-based clinical practice guidelines (CPGs). By identifying, appraising and synthesising the research evidence relevant to a particular area of clinical practice, rigorously developed CPGs can be an important part of the process by which research evidence is translated into improved practice. [4,5] Evidence-based CPGs have been demonstrated to improve both the process and the outcomes of health care delivery.[6] However, while the number of published CPGs is increasing [5,7] there is evidence from a range of settings that the quality of CPGs is often low and the link between research and recommendations is an area of particular weakness. [8-10]

Recognising the potential of CPGs to improve practice, one of the strategies of the SEA-ORCHID project was to facilitate the development (or adaptation) and implementation of evidence-based CPGs, and to support clinical staff in each of the four countries to build their skills in development of CPGs.

### Aim of this study

This study was undertaken to investigate the impact of the SEA-ORCHID project on development and adaptation of evidence-based CPGs in the nine participating hospitals in Thailand, Malaysia, Philippines and Indonesia.

## Methods

**Figure 1:**
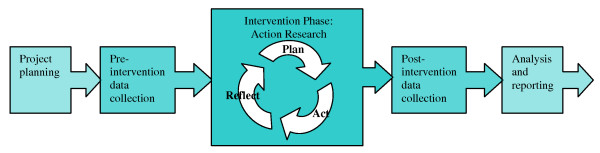
**Stages of the SEA-ORCHID project**.

The SEA-ORCHID project consists of five stages; pre-study, pre-intervention data collection, intervention, post-intervention data collection, and reporting and dissemination. Details of the project methods and pre-intervention clinical practice and health outcome data have been published previously [3,11] (Figure 1).

### Pre-intervention data collection

In 2005, prior to the implementation of the project intervention, the SEA-ORCHID team members in each of the South East Asian hospitals were asked to collect information on the nationally and locally developed guidelines relating to maternal and perinatal clinical practice which were in use in their hospitals. Standard data collection forms were used by the SEA-ORCHID team members to collect information on the nature and development methods for each of the guidelines currently used in practice. SEA-ORCHID team members were doctors and nurses working in the maternal and perinatal units of the participating hospitals. Details of the team members for each of the hospitals can be found at .

### Intervention

During the 18-month intervention phase of the project, a variety of strategies were implemented by SEA-ORCHID team members working at each of the hospitals in South East Asia, supported by the team from Australia. These strategies aimed to improve the implementation of evidence-based perinatal practice in ways that were tailored to the context and needs of each hospital.

Some of the intervention strategies of the SEA-ORCHID project were focused on improving skills in development (or adaptation) of evidence-based CPG development and implementation, and on supporting evidence-based CPG development, adaptation and implementation within the hospital quality improvement processes.

SEA-ORCHID teams ran local, national and international workshops and seminars on evidence-based CPG development and implementation, and several health practitioners from South East Asia undertook fellowships in Australia in order to have protected time to develop skills and gain practical experience in evidence-based CPG development.

Examples of activities undertaken to support CPG development & implementation within SEA-ORCHID: (Details of the strategies implemented at each of the hospitals can be found at .)

• *International workshop on CPG development held in Indonesia*

• *National symposia on CPG development held in The Philippines*

• *Fellowships on CPG development in Australia for obstetricians and neonatologists from SEA-ORCHID hospitals*

• *Workshops and training sessions on CPG development, adaptation, appraisal and implementation at each SEA-ORCHID hospital run by SEA-ORCHID Investigators, Educators and Fellows*

• *Departmental meetings within SEA-ORCHID hospitals to follow-up progress on CPG development*

• *Membership on national working groups for CPG development by SEA-ORCHID Investigators, Educators and Fellows*

### Post-intervention data collection

In 2008, after the project intervention period, SEA-ORCHID team members in each of the hospitals were asked to use standard data collection forms to update the list of the nationally and locally developed guidelines related to clinical practice in pregnancy and childbirth, noting any new guidelines or updates of existing guidelines.

Team members were also asked to identify whether these guidelines were developed on the basis of a systematic review of the available research evidence, and whether the guideline development team was multidisciplinary.

We initially planned to evaluate the rigour of development of the identified guidelines using AGREE criteria [12], however after reviewing several of the guidelines it was decided that this was not feasible since the methods of guideline development were rarely reported. From discussions with SEA-ORCHID investigators and educators it was clear that at baseline, few guidelines had been developed using rigorous evidence-based approaches.

## Results

We have not provided numbers of guidelines at each of the sites as these are very difficult to interpret; for example some hospitals had one broad protocol that covered a large number of clinical areas, whereas in other hospitals each clinical area was addressed by individual, smaller guidelines.

### Pre-intervention data collection

Prior to the intervention phase, the level of evidence-based CPG development in the SEA-ORCHID hospitals was relatively low.

In Malaysia, a few CPGs based on systematic searches for evidence and multidisciplinary involvement had been developed at a national level, and were locally implemented. The hospitals also had several of their own, non-evidence-based local protocols.

In the Indonesian hospitals practice was largely guided by the hospitals own, largely non-evidence-based local protocols. There were two nationally developed CPGs, however no CPGs were available that had been developed on the basis of systematic searches for evidence and multidisciplinary involvement.

In the Philippines, three national CPGs were available, all of which had been developed on the basis of systematic searches for evidence and two of which had included multidisciplinary involvement. A non-evidence-based book of procedures was also available to guide practice. The hospitals also had their own, non-evidence-based local protocols.

In Thailand there were several local CPGs for specific areas of practice in use at the hospitals and one regional guideline. The majority of the guidelines had been developed on the basis of systematic searches for evidence and multidisciplinary involvement. The hospitals also had their own non-evidence-based local protocols.

The topic areas addressed by national and local guidelines and protocols at baseline are provided in Additional File 1.

### Post-intervention data collection

There was relatively little evidence-based guideline development or adaptation undertaken at most of the SEA-ORCHID hospitals during the intervention period.

In the Indonesian and Malaysian hospitals no additional CPGs were in use in the clinical areas associated with SEA-ORCHID.

In The Philippines, two additional CPGs were developed at a national level, one national CPG was updated, and one CPG was developed at Philippines General Hospital. In each case these were developed in on the basis of systematic searches for evidence and multidisciplinary involvement, however these were largely driven by activities outside of the SEA-ORCHID project.

In Thailand, two new CPGs were developed on the basis of systematic searches for evidence and multidisciplinary involvement at one of the hospitals, five new CPGs were developed on the basis of systematic searches for evidence and multidisciplinary involvement at another hospital and four new CPGs developed by a multidisciplinary team, but not on the basis of systematic searches, were written at the third hospital.

## Discussion

The limited CPG development undertaken at the SEA-ORCHID hospitals during the project highlights the real, practical difficulty of developing evidence-based CPGs in hospital settings in resource-poor countries. In spite of good intentions, the availability of high quality evidence and training in evidence-based guideline development, the staff at the hospitals participating in SEA-ORCHID struggled to develop evidence-based guidelines.

Interviews with clinicians working in the SEA-ORCHID hospitals suggested that while CPGs were valued, a number of reasons why CPGs were not developed or adapted more frequently in their hospitals as is reported in detail in our exploration of the barriers to CPG development. [13] For example, the clinicians in these hospitals agreed that, as has been reported elsewhere, the accepted evidence-based CPG development process "is slow, laborious, and expensive".[4] The substantial requirements in terms of knowledge, resources, skills and time to carry out the systematic reviews of research literature required for rigorous evidence-based CPG development [5,14] are often barriers in hospital environments and this was also true in the SEA-ORCHID hospitals. Adaptation of existing evidence-based guidelines may help overcome these barriers, however adaptation was not widely undertaken in the SEA-ORCHID hospitals during the project, and the reasons for this are discussed in the companion paper to this article. [13]

While guides to the process of developing evidence-based CPGs are widely available, these have largely been produced to guide CPG development at a national level, and not in hospital settings. CPG development in a hospital setting may well require a different approach. For CPGs to be developed (or adapted) and implemented in hospital environments, like those in the SEA-ORCHID project, new strategies will need to be developed to overcome the barriers identified, and make the process more feasible.

It has recently been argued that given the wide variations in health systems, resource availability and disease epidemiology, low-income countries will increasingly need to develop guidelines locally, rather than being able to use guidelines that are developed centrally, by organisations such as WHO. [15] If this is the case, then it would further support the need for new, pragmatic but reliable evidence-based guideline development methods which make the guideline development process more feasible in environments with limited resources.

The SEA-ORCHID project was a large, complex project, in which development of CPGs was just one of several secondary outcomes. The results of this study suggest that the SEA-ORCHID teams in most hospitals chose to focus their efforts and apply the project resources on achieving changes in other project outcomes, such as clinical practice change and systematic review development, where perhaps change was perceived as easier to achieve. The results of the SEA-ORCHID project on these other outcomes will be reported elsewhere.

One of the key limitations of this research was the definition of CPG as compared to protocol or procedure. To gather the baseline and post-intervention data, we relied on self-report by SEA-ORCHID team members in each of the hospitals and it was clear that definitions of CPG, protocol and procedure, as well as what it meant to systematically search for evidence and involve a multidisciplinary team, varied between the hospitals. We do not believe that these differences would fundamentally alter the conclusions of the research, however they do make interpreting the differences between hospitals difficult. The extensive time commitment required to rigorously develop CPGs may mean that there are some CPGs for which development has begun but which are not yet completed, and therefore have not been included in the results, however we are aware of only a small number of CPGs in this situation.

## Conclusion

While there is a growing awareness of the importance of evidence-based CPGs in maternal and perinatal care in SEA-ORCHID hospitals in Thailand, Malaysia, Philippines and Indonesia, few of the currently available CPGs have been developed in a rigorously evidence-based way, and the SEA-ORCHID project did not result in a substantial increase in guideline production at most hospitals.

If development of evidence-based CPGs in hospital settings is to be increased, new strategies will need to be developed to overcome the substantial barriers and make the process more feasible.

## Competing interests

The authors declare that they have no competing interests.

## Authors' contributions

JT coordinated the data collection and analysis and provided feedback on drafts of the article. TT assisted in the data collection and analysis, prepared the first draft of the article and revised subsequent drafts. SS assisted in the data collection and provided feedback on drafts of the article. All authors read and approved the final manuscript.

## Supplementary Material

Additional file 1 **Clinical areas addressed by identified guidelines and protocols pre-intervention in the SEA-ORCHID project**. Clinical areas addressed by identified guidelines and protocols pre-intervention in the SEA-ORCHID project Click here for file
