# Supplementary material for: Maternal and perinatal guideline development in hospitals in South East Asia: results from the SEA-ORCHID project
Source: Health Res Policy Syst. 2009 May 8;7:9. doi: 10.1186/1478-4505-7-9 (PMC2683834; doi:10.1186/1478-4505-7-9)
Supplement: Additional file 1 — Clinical areas addressed by identified guidelines and protocols pre-intervention in the SEA-ORCHID project. Clinical areas addressed by identified guidelines and protocols pre-intervention in the SEA-ORCHID project [file 1478-4505-7-9-S1.doc]

**Additional File 1.** Clinical areas addressed by identified guidelines and protocols pre-intervention in the SEA-ORCHID project.

|  | Indonesia | Malaysia | The Philippines | Thailand |
| --- | --- | --- | --- | --- |
| National | - Management of hypertension in pregnancy - Intrauterine fetal growth | - Antenatal corticosteroids to prevent respiratory distress - Management of jaundice in healthy term newborns - Oxygen in the newborn - Postnatal corticosteroids for the prevention and treatment of chronic lung disease - Surfactant in the treatment of respiratory distress syndrome - Ultrasound in pregnancy | - Normal labour and delivery - Antenatal screening - Hypertension in pregnancy |  |
| Local | - Amniocentesis - Amnioscopy - Antenatal care - Asphyxia - Augmentation of labour - Blood transfusion - Breast care - Breech delivery - Caesarean section - Curretage for incomplete abortion - Eclampsia - Embriotomy - Episiotomy - External cephalic version - Fetal surveillance using cardiotocography - Forceps extraction - High risk pregnancy detection and management - Hyperemesis gavidarum - Induction of labour - Lactation management - Low birth weight baby - Manual evacuation of placenta - Mild preeclampsia - Newborn baby - Normal delivery - Perineal care - Perineal rupture - Postnatal care - Postpartum curettage - Postpartum hemorrhage - Preeclampsia - Premature rupture of the membrane - Rooming in - Threatened preterm delivery - Twin delivery - Vacuum extraction | - Aminophylline in preterm infants - Antepartum haemorrhage - Approach to the baby who does not gain weight - Augmentation of labour - Breast-feeding for NICU babies - Breech presentation - Cardiac disease in pregnancy - Diabetes in pregnancy - Induction of labour - Instrumental vaginal delivery - Long line insertion and maintenance - Mechanical ventilation of the neonate - Metabolic acidosis - Neonatal jaundice - Neonatal meningitis - Patent ductus arteriosus - Persistent pulmonary hypertension of the newborn - Positive VDRL and congenital syphilis in the newborn - Postnatal steroids for weaning from ventilation in chronic lung disease - Postpartum haemorrhage - Pregnancy induced hypertension - Premature rupture of membranes - Preterm labour - Prevention and screening for neonatal hypoglycaemia in term infant - Prevention of early-onset group b streptococcal infection - Prevention of meconium aspiration - Prolonged pregnancy - Prophylactic indomethacin - Risk factors associated with hearing loss - Surfactant - Thrombocytopenia - Transporting a sick neonate - IV prostaglandin infusion to maintain patency of ductus arteriosis in duct dependant congenital heart disease - Vaginal birth after previous caesarean section - Withdrawal of life support in NICU | - Admission criteria for the intensive maternal unit Antepartal and intrapartal fetal monitoring - Cardiac disease in pregnancy - Criteria for referral to the high risk clinic - Delivery and the second stage of labor - Emergency obstetric care after delivery of the baby - Emergency obstetric care during labor and delivery - Gestational diabetes mellitus - Guidelines for fetuses with congenital anomaly - Hydrops fetalis - Hypertension in pregnancy - Intrauterine growth retardation - Invasive procedures in obstetrics - Management of APAS - Management of pregnancies in women with normal fetus with unreliable menstrual period - Management of recurrent pregnancy loss - Monitoring labor - Oligohydramnios - Post partum care and counseling - Preparation for labor - The placenta and the third stage of labor | - Active management in third stage of labor - Antibiotic prophylaxis in caesarean section - Care during pregnancy and childbirth in Khon Kaen Province - Diabetes in pregnancy - HIV infection during pregnancy - Magnesium sulfate administration - Post term pregnancy - Postpartum haemorrhage / Uterine atony - Pregnancy induced hypertension - Prelabour rupture of membranes - Preterm labour - Thalassemia screening in pregnancy |
